# Influence of potentially harmful sucking habits on otitis media in children: a systematic review and meta-analysis

**DOI:** 10.1590/1807-3107bor-2025.vol39.115

**Published:** 2025-11-07

**Authors:** Ana Cláudia CASTRO-CUNHA, Luana Viviam MOREIRA, Isabela Costa GONÇALVES, Izabella Barbosa FERNANDES, Lucas Guimarães ABREU, Saul Martins PAIVA, Paulo Antônio MARTINS-JÚNIOR, Cristiane Baccin BENDO

**Affiliations:** (a) Universidade Federal de Minas Gerais – UFMG, School of Dentistry, Department of Pediatric Dentistry, Belo Horizonte, MG, Brazil.; (b) Universidade Federal dos Vales do Jequitinhonha e Macuri – UFVJM, School of Biological and Health Sciences, Department of Dentistry, Diamantina, MG, Brazil.

**Keywords:** Otitis Media, Child, Pacifiers, Bottle Feeding, Fingersucking

## Abstract

This study aimed to evaluate the influence of pacifier use, bottle feeding, and finger/thumb sucking on the occurrence of otitis media in children. Searches were conducted in grey literature and six databases: Web of Science, Cochrane Library, LILACS, Scopus, MEDLINE/PubMed, and EMBASE, from inception through May 2025. Observational studies investigating the association between harmful sucking habits and otitis media in newborns, infants, preschoolers, and older children were included. Risk of bias was assessed using the Joanna Briggs Institute tool. Meta-analysis results were reported as odds ratios (ORs) with 95% confidence intervals (CIs), and the certainty of evidence was also evaluated. A total of 36 studies were included, most of which were cohort studies (n = 14), with sample sizes ranging from 46 to 35,613. Eleven studies were incorporated into meta-analyses. Children who used a pacifier were 1.17 times more likely to develop otitis media (95%CI: 1.00–1.33) and 1.54 times more likely to develop acute otitis media (95%CI: 1.01–2.36) compared to those who did not use a pacifier. No significant association was found between bottle feeding and otitis media (OR = 0.83; 95%CI: 0.59–1.17). Most studies did not report a significant association between finger or thumb sucking and otitis media. The certainty of evidence was rated as very low. These findings suggest that pacifier use may increase the likelihood of developing otitis media, particularly acute otitis media, with potentially relevant implications.

## Introduction

Otitis media is a highly prevalent condition in childhood and represents a significant public health concern.^1,2^ It is among the most common reasons for pediatric medical consultations and imposes a considerable economic burden on both families and healthcare systems.^3-5^ Otitis media can also lead to lasting hearing impairment, which may adversely affect speech development and learning outcomes.^6,7^ The global incidence of acute otitis media is approximately 10.8% per year, with the highest rates observed in children under the age of four years and a subsequent decline with age.^2^

The etiology of otitis media is multifactorial, with numerous risk factors reported in the literature, including daycare attendance, exposure to tobacco smoke, and inadequate breastfeeding.^8-10^ Additionally, potentially harmful sucking habits, such as pacifier use, bottle feeding, and finger/thumb sucking, have been identified as modifiable risk factors.^11-13^ These behaviors are also associated with adverse outcomes like malocclusion.^14,15^Despite these risks, such habits remain common in early childhood.^16^ Pacifiers are widely used to soothe and comfort infants and are thought to reduce the risk of sudden infant death syndrome (SIDS).^17,18^Although a systematic review has found the evidence supporting this association to be inconclusive, the American Academy of Pediatrics currently endorses pacifier use for SIDS prevention.^19^ Finger/thumb sucking is a self-soothing behavior frequently associated with dental issues such as increased overjet.^14,20^ Bottle feeding, likewise common in infancy and early childhood,^16^ is often used as a primary or supplementary feeding method.^21^ Children who are bottle fed have been shown to experience higher rates of infectious diseases, including respiratory and gastrointestinal infections, compared to those who are breastfed.^22,23^

However, the mechanisms by which these potentially harmful sucking habits may contribute to otitis media are not fully understood. One hypothesis is that the negative pressure generated during sucking may facilitate the reflux of nasopharyngeal secretions into the middle ear.^24^ Another theory suggests that pacifier use might cause mechanical obstruction of the nasopharynx by elevating the soft palate, potentially impairing Eustachian tube function, a known contributor to otitis media.^13,25^ These habits may also indirectly influence otitis media risk by reducing breastfeeding frequency or duration, thereby limiting its protective effects against infections in infancy.^22,23,26^

Despite the clinical relevance of these behaviors, no systematic reviews to date have specifically addressed the association between otitis media and either bottle feeding or finger/thumb sucking. While one meta-analysis evaluating risk factors for otitis media included pacifier use, it was published over two decades ago and focused exclusively on acute otitis media.^8^ Given the limited and inconsistent findings in the literature, this study aimed to conduct a systematic review and meta-analysis to evaluate whether potentially harmful sucking habits, namely pacifier use, bottle feeding and finger/thumb sucking, are associated with the occurrence of otitis media in newborns, infants, preschoolers, and older children.

## Methods

This systematic review was conducted in accordance with the Preferred Reporting Items for Systematic Reviews and Meta-Analyses (Prisma) statement.^27^ The methodology was detailed in a previously published protocol article,^28^ and the protocol was registered in Prospero (CRD42020197162).

### Eligibility criteria

The review was guided by a PECOS (Population, Exposure, Comparison, Outcomes, Study design) research framework: a) Population: children aged 0 to 12 years; b) Exposure: finger/thumb sucking, pacifier use, or bottle feeding; c) Comparison: absence of finger/thumb sucking, pacifier use, or bottle feeding (exclusive breastfeeding); 4) Outcome: otitis media;^29^ 5) Study design: observational studies, including cross-sectional, case-control, and cohort designs.

The age range was determined based on Medical Subject Headings (MeSH) definitions to encompass all stages of childhood: newborns (first 28 days after birth), infants (1 to 23 months), preschool children (2 to 5 years), and children (6 to 12 years). Studies were excluded if they evaluated formula feeding without specifying whether it was provided via a bottle, or if they included children with cleft palate.

### Search strategy and information sources

The search strategy was designed to identify both published and unpublished studies. An initial limited search of the MEDLINE/PubMed database was conducted to identify relevant articles. Keywords, title and abstract terms, and MeSH terms were used to develop a comprehensive strategy. Searches were performed across six electronic databases: Web of Science, Cochrane Library, LILACS, Scopus, MEDLINE/PubMed, and EMBASE. A tailored strategy was applied to each database, as detailed in Table 1. Grey literature was searched using Google Scholar, the National Institute for Health and Care Excellence (NICE), and ProQuest Dissertations & Theses Global, with results limited to the first 300 records.^30^ All databases were searched from inception to May 2025, with no restrictions on language or publication year. Reference lists of the included studies were also screened to identify additional eligible studies. The search was updated shortly before the final analysis. Two reviewers (ACCC and LVM) were calibrated for the study selection using Fleiss’ Kappa Test, which yielded a coefficient of 0.86 based on the inclusion and exclusion criteria applied to a 20% random sample of the retrieved records.

**Table 1:** Search strategies.

Database	Search strategy
MEDLINE/PubMed	(pacifier OR pacifiers OR dummy OR dummies OR soother OR soothers OR bottlefeed OR “bottle feed” OR bottle-feed OR bottlefeeding OR “bottle feeding” OR bottle-feeding OR bottlefed OR “bottle fed” OR bottle-fed OR “nursing bottle” OR “nursing bottles” OR fingersucking OR “finger sucking” OR “finger-sucking” OR thumbsucking OR “thumb sucking” OR “thumb-sucking” OR “deleterious habit” OR “deleterious habits” OR “deleterious oral habit” OR “deleterious oral habits” OR “deleterious sucking habit” OR “deleterious sucking habits” OR “sucking habit” OR “sucking habits” OR “nonnutritive sucking habit” OR “nonnutritive sucking habits” OR “non nutritive sucking habit” OR “non nutritive sucking habits” OR “non-nutritive sucking habit” OR “non-nutritive sucking habits” OR “breast feeding” OR breastfeeding OR breast-feeding OR breastfeed OR “breast feed” OR breast-feed OR breastfed OR “breast fed” OR breast-fed OR weaning OR “sucking behavior” OR “sucking behaviors” OR “feeding behavior” OR “feeding behaviors”)
	AND (child OR children OR “preschool child” OR “preschool children” OR infant OR infants OR childhood OR toddler OR toddlers OR preschool OR preschoolers OR schoolchild OR “school child” OR schoolchildren OR “school children” OR kid OR kids OR newborn OR newborns OR youth OR youths OR pediatric OR pediatrics OR paediatric OR paediatrics OR pedodontic OR pedodontics)
	AND (otitis OR “ear inflammation” OR “ear infection” OR otitides)
LILACS	tw:((tw:(pacifier)) OR (tw:(pacifiers)) OR (tw:(dummy)) OR (tw:(dummies)) OR (tw:(soother)) OR (tw:(soothers)) OR (tw:(bottlefeed)) OR (tw:(bottle-feed)) OR (tw:(“bottle feed”)) OR (tw:(bottlefeeding)) OR (tw:(bottle-feeding)) OR (tw:(“bottle feeding”)) OR (tw:(bottlefed)) OR (tw:(bottle-fed)) OR (tw:(“bottle fed”)) OR (tw:(“nursing bottle”)) OR (tw:(“nursing bottles”)) OR (tw:(fingersucking)) OR (tw:(finger-sucking)) OR (tw:(“finger sucking”)) OR (tw:(thumbsucking)) OR (tw:(thumb-sucking)) OR (tw:(“thumb sucking”)) OR (tw:(“deleterious habit”)) OR (tw:(“deleterious habits”)) OR (tw:(“deleterious oral habit”)) OR (tw:(“deleterious oral habits”)) OR (tw:(“deleterious sucking habit”)) OR (tw:(“deleterious sucking habits”)) OR (tw:(“sucking habit”)) OR (tw:(“sucking habits”)) OR (tw:(“nonnutritive sucking habit”)) OR (tw:(“nonnutritive sucking habits”)) OR (tw:(“non nutritive sucking habit”)) OR (tw:(“non nutritive sucking habits”)) OR (tw:(“non-nutritive sucking habit”)) OR (tw:(“non-nutritive sucking habits”)) OR (tw:(“breast feed”)) OR (tw:(breastfeed)) OR (tw:(breast-feed)) OR (tw:(“breast feeding”)) OR (tw:(breastfeeding)) OR (tw:(breast-feeding)) OR (tw:(“breast fed”)) OR (tw:(breastfed)) OR (tw:(breast-fed)) OR (tw:(weaning)) OR (tw:(“sucking behavior”)) OR (tw:(“sucking behaviors”)) OR (tw:(“feeding behavior”)) OR (tw:(“feeding behaviors”)))
	AND tw:((tw:(child)) OR (tw:(children)) OR (tw:(“preschool child”)) OR (tw:(“preschool children”)) OR (tw:(infant)) OR (tw:(infants)) OR (tw:(childhood)) OR (tw:(toddler)) OR (tw:(toddlers)) OR (tw:(preschool)) OR (tw:(preschoolers)) OR (tw:(schoolchild)) OR (tw:(“school child”)) OR (tw:(schoolchildren)) OR (tw:(“school children”)) OR (tw:(kid)) OR (tw:(kids)) OR (tw:(newborn)) OR (tw:(newborns)) OR (tw:(youth)) OR (tw:(youths)) OR (tw:(pediatric)) OR (tw:(pediatrics)) OR (tw:(paediatric)) OR (tw:(paediatrics)) OR (tw:(pedodontic)) OR (tw:(pedodontics)))
	AND tw:((tw:(otitis)) OR (tw:(“ear inflammation”)) OR (tw:(“ear infection”)) OR (tw:(otitides)))
Scopus	TITLE-ABS-KEY (pacifier OR pacifiers OR dummy OR dummies OR soother OR soothers OR bottlefeed OR “bottle feed” OR bottle-feed OR bottlefeeding OR “bottle feeding” OR bottle-feeding OR bottlefed OR “bottle fed” OR bottle-fed OR “nursing bottle” OR “nursing bottles” OR fingersucking OR “finger sucking” OR “finger-sucking” OR thumbsucking OR “thumb sucking” OR “thumb-sucking” OR “deleterious habit” OR “deleterious habits” OR “deleterious oral habit” OR “deleterious oral habits” OR “deleterious sucking habit” OR “deleterious sucking habits” OR “sucking habit” OR “sucking habits” OR “nonnutritive sucking habit” OR “nonnutritive sucking habits” OR “non nutritive sucking habit” OR “non nutritive sucking habits” OR “non-nutritive sucking habit” OR “non-nutritive sucking habits” OR “breast feeding” “breastfeeding” “breast-feeding” OR breastfeed OR “breast feed” OR breast-feed OR breastfed OR “breast fed” OR breast-fed OR weaning OR “sucking behavior” OR “sucking behaviors” OR “feeding behavior” OR “feeding behaviors”)
	AND TITLE-ABS-KEY (child OR children OR “preschool child” OR “preschool children” OR infant OR infants OR childhood OR toddler OR toddlers OR preschool OR preschoolers OR schoolchild OR “school child” OR schoolchildren OR “school children” OR kid OR kids OR newborn OR newborns OR youth OR youths OR pediatric OR pediatrics OR paediatric OR paediatrics OR pedodontic OR pedodontics)
	AND TITLE-ABS-KEY (otitis OR “ear inflammation” OR “ear infection” OR otitides)
Web of Science	TS=(pacifier OR pacifiers OR dummy OR dummies OR soother OR soothers OR bottlefeed OR “bottle feed” OR bottle-feed OR bottlefeeding OR “bottle feeding” OR bottle-feeding OR bottlefed OR “bottle fed” OR bottle-fed OR “nursing bottle” OR “nursing bottles” OR fingersucking OR “finger sucking” OR “finger-sucking” OR thumbsucking OR “thumb sucking” OR “thumb-sucking” OR “deleterious habit” OR “deleterious habits” OR “deleterious oral habit” OR “deleterious oral habits” OR “deleterious sucking habit” OR “deleterious sucking habits” OR “sucking habit” OR “sucking habits” OR “nonnutritive sucking habit” OR “nonnutritive sucking habits” OR “non nutritive sucking habit” OR “non nutritive sucking habits” OR “non-nutritive sucking habit” OR “non-nutritive sucking habits” OR “breast feeding” OR “breastfeeding” OR “breast-feeding” OR breastfeed OR “breast feed” OR breast-feed OR breastfed OR “breast fed” OR breast-fed OR weaning OR “sucking behavior” OR “sucking behaviors” OR “feeding behavior” OR “feeding behaviors”)
	AND TS=(child OR children OR “preschool child” OR “preschool children” OR infant OR infants OR childhood OR toddler OR toddlers OR preschool OR preschoolers OR schoolchild OR “school child” OR schoolchildren OR “school children” OR kid OR kids OR newborn OR newborns OR youth OR youths OR pediatric OR pediatrics OR paediatric OR paediatrics OR pedodontic OR pedodontics)
	AND TS=(otitis OR “ear inflammation” OR “ear infection” OR otitides)
Cochrane Library	#1 pacifier; #2 MeSH descriptor: pacifiers; #3 dummy; #4 dummies; #5 soother; #6 soothers; #7 bottlefeed; #8 “bottle feed”; #9 bottle-feed; #10 bottlefeeding; #11 MeSH descriptor: “bottle feeding”; #12 bottle-feeding; #13 bottlefed; #14 “bottle fed”; #15 bottle-fed; #16 “nursing bottle”; #17 “nursing bottles”; #18 MeSH descriptor: fingersucking; #19 “finger sucking”; #20 “finger-sucking”; #21 thumbsucking; #22 “thumb sucking”; #23 “thumb-sucking”; #24 “deleterious habit”; #25 “deleterious habits”; #26 “deleterious oral habit”; #27 “deleterious oral habits”; #28 “deleterious sucking habit”; #29 “deleterious sucking habits”; #30 “sucking habit”; #31 “sucking habits”; #32 “nonnutritive sucking habit”; #33 “nonnutritive sucking habits”; #34 “non nutritive sucking habit”; #35 “non nutritive sucking habits”; #36 “non-nutritive sucking habit”; #37 “non-nutritive sucking habits”; #38 MeSH descriptor: “breast feeding”; #39 “breastfeeding”; #40 “breast-feeding”; #41 breastfeed; #42 “breast feed”; #43 breast-feed; #44 breastfed; #45 “breast fed”; #46 breast-fed; #47 MeSH descriptor: weaning; #48 MeSH descriptor: “sucking behavior”; #49 “sucking behaviors”; #50 MeSH descriptor: “feeding behavior”; #51 “feeding behaviors”
	#53 MeSH descriptor: child; #54 children; #55 “preschool child”; #56 “preschool children”; #57 MeSH descriptor: infant; #58 infants; #59 childhood; #60 toddler; #61 toddlers; #62 MeSH descriptor: preschool; #63 preschoolers; #64 schoolchild; #65“school child”; #66 schoolchildren; #67 “school children”; #68 kid; #69 kids; #70 MeSH descriptor: newborn; #71 newborns; #72 youth; #73 youths; #74 pediatric; #75 MeSH descriptor: pediatrics; #76 paediatric; #77 paediatrics; #78 pedodontic; #79 pedodontics
	#81 MeSH descriptor: otitis; #82 “ear inflammation”; #83 “ear infection”; #84 otitides
	#52 AND #80 AND #85
EMBASE	(pacifier OR pacifiers OR dummy OR dummies OR soother OR soothers OR bottlefeed OR “bottle feed” OR bottle-feed OR bottlefeeding OR “bottle feeding” OR bottle-feeding OR bottlefed OR “bottle fed” OR bottle-fed OR “nursing bottle” OR “nursing bottles” OR fingersucking OR “finger sucking” OR “finger-sucking” OR thumbsucking OR “thumb sucking” OR “thumb-sucking” OR “deleterious habit” OR “deleterious habits” OR “deleterious oral habit” OR “deleterious oral habits” OR “deleterious sucking habit” OR “deleterious sucking habits” OR “sucking habit” OR “sucking habits” OR “nonnutritive sucking habit” OR “nonnutritive sucking habits” OR “non nutritive sucking habit” OR “non nutritive sucking habits” OR “non-nutritive sucking habit” OR “non-nutritive sucking habits” OR “breast feeding” OR breastfeeding OR breast-feeding OR breastfeed OR “breast feed” OR breast-feed OR breastfed OR “breast fed” OR breast-fed OR weaning OR “sucking behavior” OR “sucking behaviors” OR “feeding behavior” OR “feeding behaviors”)
	AND (child OR children OR “preschool child” OR “preschool children” OR infant OR infants OR childhood OR toddler OR toddlers OR preschool OR preschoolers OR schoolchild OR “school child” OR schoolchildren OR “school children” OR kid OR kids OR newborn OR newborns OR youth OR youths OR pediatric OR pediatrics OR paediatric OR paediatrics OR pedodontic OR pedodontics)
	AND (otitis OR “ear inflammation” OR “ear infection” OR otitides)
Google Scholar	child OR infant OR newborn AND pacifier OR dummy OR thumb OR finger OR bottle OR sucking OR breast OR “oral habit” AND otitis
ProQuest	child OR infant OR newborn AND pacifier OR dummy OR thumb OR finger OR bottle OR sucking OR breast OR “oral habit” AND otitis
National Institute for Health and Care Excellence	child OR infant OR newborn AND pacifier OR dummy OR thumb OR finger OR bottle OR sucking OR breast OR “oral habit” AND otitis

### Study selection

**Figure 1:**
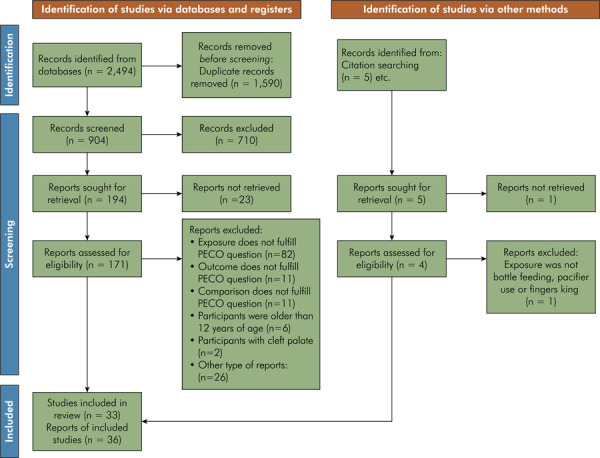
Flowchart of study selection.

All references retrieved during the searches were exported to Endnote Web (Clarivate Analytics, Philadelphia, USA) for duplicate identification and removal. A secondary manual review was conducted to identify any remaining duplicates. Study selection was performed independently by two reviewers (ACCC and LVM), with disagreements resolved through consultation with a third reviewer (IBF). Titles and abstracts were screened, and articles that appeared to meet the eligibility criteria were preselected for full-text analysis. If the title or abstract did not provide sufficient information to determine eligibility, the full text was retrieved for evaluation. All studies that met the inclusion criteria during full-text review were included in the systematic review. The study selection process is depicted in a flowchart in the Results section (Figure 1), in accordance with Prisma guidelines.^27^

### Data extraction

Data from all included studies were extracted independently by two reviewers (ACCC and LVM) and organized in a Microsoft Excel® spreadsheet (Microsoft Corporation, Redmond, USA). The following information was collected: first author’s last name, year of publication, journal, country where the study was conducted, sample size, age of participants, types of potentially harmful sucking habits evaluated (pacifier use, bottle feeding, and finger/thumb sucking), types of otitis media assessed (acute otitis media, recurrent otitis media, and otitis media with effusion), methods of otitis media assessment (parental report, medical records, or clinical diagnosis), and key findings on the association between potentially harmful sucking habits and otitis media (p-values, odds ratios, and confidence intervals).

### Assessment of risk of bias

The risk of bias in the included studies was independently assessed by two reviewers (ACCC and LVM) using the Joanna Briggs Institute (JBI) Critical Appraisal Tool developed by the University of Adelaide.^31^ Any disagreements were resolved through discussion with a third reviewer (IBF).

The risk of bias in cross-sectional studies was evaluated using the following eight criteria: a) clearly defined inclusion criteria; b) appropriate sample size; c) detailed participant characteristics; d) description of study setting and time point; e) use of valid and reliable methods to assess exposure; f) application of objective and standard criteria for outcome assessment; g) sufficient coverage of data analysis; h) appropriate handling of response rates and use of suitable statistical analysis.

The risk of bias in case-control studies was assessed using eleven criteria: a) similarity between groups; b) confirmed presence of the condition in cases and absence in controls; c) appropriate matching of cases and controls; d) consistent criteria used to identify cases and controls; e) use of a valid and reliable method to measure exposure; f) consistent measurement of exposure for both cases and controls; g) identification of confounding factors; h) strategies to address confounding factors; i) use of a valid and reliable method for outcome assessment in both groups; j) evaluation of the exposure period and whether it was sufficiently long to be meaningful; and k) use of appropriate statistical analysis.

The risk of bias in cohort studies was evaluated based on 12 criteria: a) recruitment of participants from the same population for both groups; b) consistent measurement of exposure to assign participants to exposed and unexposed groups; c) use of a valid and reliable instrument to assess exposure; d) identification of confounding variables; e) strategies to manage confounding variables; f) confirmation that participants were free of the outcome at baseline; g) use of a valid and reliable method for outcome assessment; h) clear reporting of the outcomes; i) specification of the follow-up period and its adequacy for outcome development; j) information on whether follow-up was complete or reasons for loss to follow-up were provided; k) strategies to address incomplete follow-up; and l) use of appropriate statistical analysis.

Each item in each study was assigned one of four risk classifications: “low risk of bias” (if the response was “Yes”), “high risk of bias” (if “No”), “unclear risk of bias” (if the information was not clearly stated or not found in the article), and “not applicable” (if the item did not pertain to the study type). A study was considered at low risk of bias if otitis media was explicitly diagnosed using otoscopy, tympanometry, or by isolating an otitis-related pathogen in a culture, as part of outcome assessment. If the diagnosis was based solely on parental report or extracted from medical records, the risk of bias was considered high, even in cases where an episode was reported to have been diagnosed by a physician.

### Synthesis of results

Meta-analyses were conducted using Review Manager software (RevMan, version 5.4; The Cochrane Collaboration, 2020). Generic inverse variance meta-analyses were performed. When available, effect estimates (ratio measures) and their standard errors (SEs) were calculated directly into RevMan. For ratio measures, the data were entered as natural logarithms. SEs were calculated by subtracting the lower limit from the upper limit of the confidence interval (CI) and dividing the result by 3.92.^32^ In studies presenting dichotomous data, effect estimates and corresponding CIs were calculated using 2 × 2 contingency tables,^33^with MedCalc software (version 19.2.6; MedCalc Software bv, Ostend, Belgium; https://www.medcalc.org; 2020). In meta-analyses where studies with different designs were pooled, subgroup analyses were conducted by study design. Heterogeneity was assessed using the I^2^ statistic in all meta-analyses. I^2^ values were interpreted as follows: 0%–40%: may not be important; 30%–60%: may represent moderate heterogeneity; 50%–90%: may represent substantial heterogeneity; and 75%–100%: considerable heterogeneity.^32^

The magnitude of association (effect size, ES) was also calculated. ES was determined for each meta-analysis by dividing the natural logarithm of the odds ratio by 1.81. The SE of the ES was calculated by transforming the CI of the odds ratio into an SE and dividing the result by 1.81. A full description of this method is available elsewhere.^34^ An ES of approximately 0.20 was considered small, an ES close to 0.5 moderate, and an ES close to 0.8 large.^35^

### Assessment of certainty of evidence

The certainty of the evidence was assessed using the Grading of Recommendations, Assessment, Development, and Evaluation (GRADE) approach.^36^ The GRADEpro GDT software was used to evaluate certainty and to generate the summary of findings table. The following domains were considered: risk of bias, inconsistency, indirectness, imprecision, and publication bias. Based on these domains, the certainty of the evidence was classified as high, moderate, low, or very low. The outcomes considered in this analysis included otitis media overall, as well as the subgroups acute otitis media and recurrent otitis media (three or more episodes within six months).

## Results

### Study selection

The database searches yielded 2,494 references, of which 1,590 were duplicates and subsequently removed. The remaining 904 records were screened independently by two reviewers, resulting in 194 studies selected for full-text analysis. Twenty-three full texts could not be retrieved, and 138 studies were excluded following application of the eligibility criteria. Additionally, five references were identified by hand searching the reference lists of included studies, three of which met the eligibility criteria. In total, 36 studies were included in the present systematic review, 11 of which were incorporated into meta-analyses (Figure 1). Some articles were considered “near misses” because they assessed otitis media in breastfed versus formula-fed children but did not specify whether the formula was administered via a bottle. Furthermore, certain studies comparing bottle-fed and breastfed children used non-exclusive definitions: children classified as bottle-fed received only bottle feeding, whereas the breastfed group included any breastfeeding, regardless of supplementation. Since these studies did not meet the eligibility criteria, they were excluded from the present review.

### Study characteristics

The characteristics of the included studies are presented Table 2. Eleven studies had a cross-sectional design,^11-13,37-44^ eleven were case-control studies,^45-55^; and 14 were cohort studies.^23,26,56-67^ Most studies were published in English (n = 33), two were in Spanish,^39,40^ and one was in Korean.^46^ Seven studies were published before 1990;^26,41,43,44,56,57,67^ nine between 1990 and 2000,^11-13,23,55,60,64-66^; six between 2000 and 2010,^39,46,50,51,61,63^ and 14 after 2010.^37,38, 40,42,45;47-49,52-54,58,59,62^ Sample sizes ranged from 46^65^ to 35,613.^58^ Participant ages ranged from newborns to 12 years.

**Table 2:** Characteristics of included studies.

Author/Year/Country/Journal	Study design	Setting	Sampling method	Type of sucking habit	Otitis media assessment	Statistical analysis	Main outcomes
Alexandrino et al., 2016^38^; Portugal; Fam Pract	Cross-sectional	Private day care centers	152 children aged 4–36 months	Pacifier use	Acute otitis media; parental report.	No numerical data presented	No data related to pacifier use was presented.
Alho et al., 1993^64^; Finland; Arch Otolaryngol Head Neck Surg	Cohort	Primary health care centers, hospital, and private clinics	2,512 children were followed up from birth to 24 months	Bottle feeding	Acute otitis media; medical records.	No numerical data presented	No association was found between AOM and bottle feeding. No numerical data was presented.
Aliboni, 2002^39^; Argentina; Arch Argent Pediatr	Cross-sectional	Health care centers and private clinics	168 children aged 6 months to 6 years	Pacifier use	Acute otitis media; parental report.	Chi-square test	No association was found between otitis media and pacifier use (*p* = 0.072).
							62.0% of children used a pacifier; 89.2% were bottle fed. 33.0% of non–pacifier users had a finger-sucking habit.
Alpízar-Becil, 2011^40^; Cuba; Mediciego	Cross-sectional	Open Neonatology Service	191 newborns	Bottle feeding; pacifier use	Acute otitis media; medical records.	Descriptive analysis (frequency)	Among children with AOM, 15.0% used a pacifier and 17.0% were bottle fed.
Bass & Groer, 1996^65^; USA; J Perinat Neonat Nurs	Cohort	Women enrolled in WIC program were contacted by letter; if no response, they were contacted by phone, at WIC clinic visits, or through home visits.	46 newborns were followed up to 2 months of age	Bottle feeding	Ear infection; parental report.	Descriptive analysis (frequency)	At 1 month: 4.8% of “all formula” and 16.7% of “breast and bottle feeding” children had ear infections. At 2 months: 12.0% of “all formula” and 2.0% of “breast and bottle feeding” children had ear infections.
Engel et al., 1999^66^; Netherlands; Int. J. Pediatr. Otorhinolaryngol.	Cohort	Hospital	192 newborns were followed up to 2 years of age	Bottle feeding	Otitis media with effusion; parental report.	No numerical data presented	No data related to bottle feeding were presented.
Forman et al., 1994^41^; USA; Int J Epidemiol	Cross-sectional	Hospital and medical clinic	571 infants under 18 months	Bottle feeding	Otitis media; medical records.	Two-way tabulations; chi-square test; multivariable logistic regression	From birth to four months: exclusively breastfed children had fewer URTI + otitis cases than exclusively bottle-fed (χ^2^ = 4.07, *p* = 0.05) and mixed-fed children (χ^2^ = 10.07, *p* = 0.005).
							No significant differences were observed among feeding groups beyond the first four months of life.
Fosarelli et al., 1985^67^; USA; Pediatr Infect Dis	Cohort	Primary health care clinic	279 children under 12 months of age	Bottle feeding	Otitis media; medical records.	Chi-square test	Breastfed children had fewer episodes of otitis media than bottle-fed children (*p* <0.05).
Frank et al., 1982^56^; USA; Pediatrics	Cohort	Hospital	First semester: 81 infants, Second semester: 68 infants. Infants under 12 months of age.	Bottle feeding	Otitis media; physician diagnosis	Descriptive analysis (frequency and rate)	In the first semester, 3 cases of otitis media occurred among 42 bottle-fed children (rate = 7); in the second semester, 15 cases occurred among 36 bottle-fed children (rate = 42).
Giuca et al., 2011^43^; Itália; Eur Arch Paediatr Dent	Case-control	Patients were referred from the Department of Otolaryngology	50 children with a mean age of 7.8 ± 1.0 years.	Thumb sucking	Recurrent otitis media;	Pearson’s chi-square test/Fisher’s exact test	No association was found between thumb sucking and otitis media (*p* = 1).
Hardani et al., 2020^47^, Iran	Case-control	Hospital	530 children were examined. After ear examination, 106 children (53 cases and 53 controls) aged 6 months to 7 years were included.	Use of a pacifier or bottle	Otitis media (acute, chronic, and with effusion); physician diagnosis based on otoscopic examination and tympanometry.	Chi-square test; multivariable logistic regression	Among the children with otitis media, 73.6% had a history of pacifier or bottle use, which is a significantly higher rate than in the control group (*p* = 0.000). Logistic regression: OR = 0.156; 95% CI: 0.05–0.58; *p* = 0.000.
Holmes et al., 1983^57^; USA; Pediatrics	Cohort	University Medical Center	251 newborns were followed up to 12 months of age.	Bottle feeding	Acute otitis media; medical records and patient-reported history.	Descriptive analysis (frequency)	Nine cases of AOM occurred among bottle-fed children, versus three among breastfed children (2 in ≤ 3-months subgroup, and 1 in > 3-months subgroup).
Howie et al., 1990^23^; Scotland; Br Med J	Cohort	Hospital and home visits	618 newborns were followed up to 24 months of age.	Bottle feeding	Ear infection; parental report and medical records.	Odds ratio and 95% CI	No statistical difference was found between bottle and breastfeeding for ear infection; partial breastfeeding was included in the breastfed group.
Jackson & Mourino, 1999^11^; USA; Pediatr Dent	Cross-sectional	Hospital	200 children under 12 months of age	Bottle feeding; pacifier use (5 or more hours); thumb sucking	Otitis media; medical records.	Pearson’s chi-square test	Association found between otitis media and bottle feeding (*p* = 0.05; OR = 5.08).
							Association found between otitis media and pacifier use (*p* = 0.04; OR = 2.09).
							Association found between otitis media and thumb sucking (*p* = 0.02; OR = 0.45).
Kim et al., 2010^46^; South Korea; Korean J Pediatr Infect Dis	Case-control	Hospital	435 children under 60 months of age	Pacifier use	Recurrent otitis media; parental report.	Chi-square test; multivariable logistic regression	No association found between pacifier use and ROM (*p* = 0.52; OR = 1.17; 95% CI: 0.7214–1.91).
Korvel-Hanquist et al., 2018^58^; Denmark; Int J Pediatr Otorhinolaryngol	Cohort	Data were extracted from the Danish National Birth Cohort. Participants were contacted by phone.	35,613 mother–child pairs (followed up at both 18 months and 7 years), plus 18,936 followed up only at 18 months, and 14,668 only at 7 years. Newborns were followed up at 6 and 18 months and/or 7 years of age.	Pacifier use	Otitis media; parental report.	Chi-square test	No association found between pacifier use and the risk of OM (*p* = 0.0133).
Lee et al., 2018^48^, South Korea; Yeungnam Univ J Med	Case-control	Hospital	57 children under 12 years old.	Bottle feeding	Otitis media with effusion; physician diagnosis.	Chi-square test; multivariable logistic regression	No association found between bottle feeding and ventilation tube insertion for OME (*p* = 0.599; OR = 1.51; 95% CI: 0.53–4.27).
Megged et al., 2018^49^; Israel; Clin Pediatr	Case-control	Medical center	163 infants under 30 days of life. Follow-up age ranged from 2 to 9 years old.	Pacifier use	Acute otitis media and recurrent acute otitis media; medical records and parental report.	Chi-square test	No association found between pacifier use and AOM (*p* = 0.4).
Morin et al., 2012^59^; Canada; BMC Pediatrics	Cohort	Community-based organizations offering recreational activities	64 children aged 6–18 months. Follow-up age ranged from 8 to 11 months.	Pacifier use	Acute otitis media; parental report.	Chi-square test; hierarchical linear and nonlinear model	Association found between pacifier use and AOM (*p* < 0.01; RR = 2.59; 95% CI: 1.51–3.22).
Mumtaz et al., 2009^50^; Pakistan; J Dow Univ Health Sci	Case-control	Hospital	128 babies under 24 months of age.	Bottle feeding	Acute otitis media; physician diagnosis.	Chi-square test	Mixed feeding (bottle + breast) was associated with otitis media (*p* = 0.003; OR = 3.0; 95% CI: 1.43–6.25).
Nery et al., 2010^51^; Brazil; Eur J Paed Dent	Case-control	University clinic	100 children (52 cases and 48 controls) aged 4–10 years.	Bottle feeding; pacifier use; thumb sucking	Otitis media with effusion; physician diagnosis.	Pearson’s chi-square test	No association found between bottle feeding and OME (*p* = 0.64; χ^2^ = 0.21; df = 1). No association found between inadequate oral habits (pacifier and thumb sucking) and OME (*p* = 1.20; χ^2^ = 1.60; df = 1).
Niemela et al., 1994^13^; Finland; Int J Pediatr Otorhinolaryngol	Cross-sectional	Municipal dental health center	938 children aged 5 years.	Pacifier use; thumb sucking	Acute otitis media; parental report.	Bivariate analysis – chi-square; multivariable logistic regression	Association found between pacifier use and AOM (RR = 1.48; *p* = 0.01; 95% CI: 1.08–2.02). No association found between thumb sucking and AOM.
Niemela et al., 1995^60^; Finland; Pediatrics	Cohort	Schools	845 children with a mean age of 3.29 years (range: 0.25–7.24 years).	Bottle feeding; pacifier use; thumb sucking	Acute otitis media and recurrent acute otitis media; parental report.	Mantel-Haenszel test; multivariable logistic regression	Pacifier use was associated with more AOM recurrences in children aged 2–3 years (test for trend χ^2^ = 6.0; *p* = 0.01); a nonsignificant trend was found in those under 2 years (test for trend χ^2^ = 2.9; *p* = 0.09). No association was found between thumb sucking and AOM (*p* = 0.32) or between bottle feeding and AOM (no *p-*value provided).
Pawathil & Rajamma, 2016^52^; India; Int J Sci Study	Case-control	University clinic	200 children under 5 years of age.	Bottle feeding	Acute otitis media; assessment method not reported.	Not reported	Bottle feeding increased AOM risk by 2.45 times.
Ralli et al., 2011^53^; Italy; Int J Pediatr Otorhinolaryngol	Case-control	University clinic	125 children (65 cases and 60 controls) aged 7–12 years.	Deleterious sucking habits (dummy sucking, digit sucking (thumb or finger sucking), and bottle feeding)	Otitis media with effusion; physician diagnosis.	Chi-square test	Deleterious sucking habits were significantly more frequent in the study group than in the control group ((*p* = 0.0457; 28/65 vs. 12/60).
Rovers et al., 2008^61^; Netherlands; Family Practice	Cohort	Data were collected from the Utrecht Health Project.	476 children aged 0–4 years.	Pacifier use	Acute otitis media and recurrent otitis media; medical records.	Logistic regression; sensitivity analysis	No statistically significant association was found between pacifier use and AOM (OR = 1.3; 95% CI: 0.9–1.9) or recurrent AOM (OR = 1.9; 95% CI: 1.1– 3.2).
Saarinen, 1982^26^, Finland; Acta Pediatr Scand	Cohort	Hospital	178 newborns followed up at 1 and 3 years of age.	Bottle feeding	Recurrent otitis media; physician diagnosis and parental report.	Chi-square test with Yates’ correction	Ages 0–1 year: longer breastfeeding vs. bottle feeding (*p* < 0.05); ages 0–3 years: longer breastfeeding vs. bottle feeding (*p* < 0.05), and intermediate breastfeeding vs. bottle (*p* < 0.10).
Saim et al., 1997^12^; Malaysia; Int J Pediatr Otorhinolaryngol	Cross-sectional	Preschool	1,097 children aged 5–6 years.	Bottle feeding	Otitis media with effusion; physician diagnosis.	Not reported	Among 639 bottle-fed children, 103 children had OME; among 458 breastfed children, 48 had OME (*p* < 0.01).
Sangeetha et al., 2014^42^; India; Al Ameen J Med Sci	Cross-sectional	Hospitals, university medical clinics (ENT departments), and university dental clinics (pediatric departments)	20 children aged 0–5 years.	Bottle feeding (bottle + breast and bottle only); pacifier use	Otitis media; physician diagnosis and medical records.	No numerical data presented	No data related to pacifier use or bottle feeding were presented.
Schaefer, 1971^43^; Canada; Can J Public Health	Cross-sectional	Not reported	184 children aged 0–9 years.	Bottle feeding	Otitis media (recurrent and chronic middle ear disease); physician diagnosis.	Not reported	Association found between otitis media and feeding mode (*p* < 0.001).
Shaw et al., 1981^44^; USA; Public Health Rep	Cross-sectional	Data on births and otitis media were collected from a service unit. Home visits were conducted at 6 weeks of age.	1,349 newborns with feeding mode data collected.	Bottle feeding (bottle + breast and only bottle)	Acute suppurative otitis media; medical records.	Descriptive analysis (frequency)	No data testing for breast vs. bottle feeding was provided because the number of non–bottle-fed infants was too small for analysis.
							Breast + bottle-fed children did not have significantly lower otitis media rates compared to exclusively bottle-fed children.
Torretta et al., 2013^54^; Italy; Int J Pediatr Otorhinolaryngol	Case-control	Telephone interviews of children treated at a medical clinic.	57 children aged 21–29 months.	Continuous pacifier use	Recurrent acute otitis media; medical records.	Fisher’s exact test	No significant association was found between pacifier use and history of RAOM (no *p*-value presented; OR = 0.90; 95% CI: 0.30–2.71).
Strutt et al., 2021^37^; UK	Cross-sectional	Nurseries and playgroups	90 children aged 24–61 months.	Pacifier use	Ear infection; parental report (questionnaire)	Poisson regression	Among 42 non–dummy users, 6 had at least one prior ear infection; among 48 dummy users, 12 had at least one prior infection. The rate was 25.0% in dummy users and 14.0% in non–dummy users, indicating a 1.8-fold increased risk in the dummy user group.
Walsh et al., 2014^62^; USA; PeerJ	Cohort	Hospital	391 children under 12 months of age were followed up by telephone at 3 and 12 months.	Pacifier use	Otitis media and recurrent otitis media; parental report.	Fisher’s exact test; multivariable regression	No significant difference in otitis media was observed between “never-users” and “pacifier users” (*p* = 0.808); no significant difference in recurrent otitis media either (*p* = 0.463).
Warren et al., 2001^63^; USA; Ped Dentistry	Cohort	Hospital	1,375 newborns were followed up at 6 weeks, and at 3, 6, 9, and 12 months.	Pacifier use; digit sucking (thumb or finger sucking)	Otitis media; parental report.	Bivariate analyses: chi-square test; multivariable analysis using generalized estimating equations (GEE)	Pacifier use was a risk factor for OM (*p* = 0.019; OR = 1.20; 95% CI: 1.03–1.39).
							No association found between digit sucking and otitis media.
Williamson et al., 1994^55^; UK; Family Practice	Case-control	Schools	265 children aged 5–7 years	Bottle feeding	Otitis media with effusion; physician diagnosis.	Odds ratio and 95% CI	Significant association found between bottle feeding and OME (OR = 2.09; 95% CI: 1.23–3.53).

AOM: Acute otitis media; OM: Otitis media; OME: Otitis media with effusion; RAOM: Recurrent acute otitis media; ROM: Recurrent otitis media.

Otitis media was diagnosed using one of three methods: parental report,^13,23,26,37-39,46,49,57-60,62,63,65,66^ information retrieved from medical records or files,^11,23,40^; and medical diagnosis by a physician.^12,26,42,43,45,47,48,50,51,53,55,56^ One study did not report how otitis media was assessed.^52^ In studies involving physician diagnosis, the primary examinations performed were otoscopy and tympanometry. Fourteen studies assessed acute otitis media,^13,38-40,44,47,49,50,52,57,59-61,64^ nine assessed recurrent otitis media,^26,43,45,46,49,54,60-62^ seven assessed otitis media with effusion,^12,47,48,51,53,55,66^ and 12 did not specify the type of otitis beyond describing it as “otitis media”^11,41,42,56,58,62,63,67^ or “ear infection.”^23,37,65^

A total of 21 studies investigated bottle feeding,^11,12,23,26,40-44,48,50-52,55-57,60,64-67^ and many of these reported a positive association with otitis media.^11,12,26,41,43,51,52,55,67^ Four studies assessed bottle feeding but did not report testing for its association with otitis media.^48,51,60,64^

Pacifier use was assessed in 17 studies.,^11,13,37-40,42,46,49,51,54,58-63^ Six studies reported an association between pacifier use and otitis media,^11,13,37,59,60,63^ while eight found no association.^39,46,49,51,54,58,61,62^ One study provided only descriptive analysis,^40^ and two others proposed assessing this variable but did not report the results.^38,42^Finger or thumb sucking was investigated in five studies,^11,13,45,60,63^. Four of these found no association with otitis media,^13,45,60,63^ and only one study reported a statistically significant association.^11^

Three studies analyzed multiple potentially harmful sucking habits as a single composite variable.^47,51,53^ Ralli et al.^53^ evaluated bottle feeding, pacifier use, and finger/thumb sucking under the category “deleterious sucking habits” and found a significant association with otitis media. Hardani et al.^47^ combined bottle feeding and pacifier use under the label “use of a pacifier or bottle,” which was also associated with otitis media. In contrast, Nery et al.^51^ grouped pacifier use and finger/thumb sucking as “inadequate oral habits” and found no association with otitis media.

### Risk of Bias

The results of the risk of bias assessment for the included studies are summarized as follows: Table 3 presents the findings for cross-sectional studies, Table 4 for case-control studies, and Table 5 for cohort studies. Most studies presented a high risk of bias regarding the outcome assessment. Twelve studies diagnosed otitis media based on physician assessment; however, only nine of these studies were classified as having a low risk of bias.^12,43,45,47,48,50,51,53,55^ Two studies combined physician diagnosis with other methods for identifying otitis media,^26,42^ and one study did not specify the diagnostic method used.^56^

**Table 3:** Assessment of methodological quality of cross-sectional studies.

Author/ Year	Clear definition of inclusion criteria	Detailed description of subjects and setting	Valid and reliable exposure measurement	Objective and standard measurement of the condition	Identification of confounding factors	Strategies to address confounding factors	Valid and reliable measurement of the outcome	Appropriate statistical analysis
Alexandrino et al., 2016^38^	High risk of bias	High risk of bias	Low risk of bias	Not applicable	Low risk of bias	Low risk of bias	High risk of bias	Low risk of bias
Aliboni, 2002^39^	Low risk of bias	Low risk of bias	Low risk of bias	Not applicable	High risk of bias	High risk of bias	High risk of bias	High risk of bias
Alpízar-Becil, 2011^40^	Low risk of bias	High risk of bias	Low risk of bias	Not applicable	Low risk of bias	High risk of bias	High risk of bias	High risk of bias
Forman et al., 1994^41^	Unclear risk of bias	Low risk of bias	Low risk of bias	Not applicable	High risk of bias	High risk of bias	High risk of bias	Low risk of bias
Jackson & Mourino, 1999^11^	Low risk of bias	High risk of bias	Low risk of bias	Not applicable	High risk of bias	High risk of bias	High risk of bias	Low risk of bias
Niemela et al., 1994^13^	Low risk of bias	High risk of bias	Low risk of bias	Not applicable	High risk of bias	Low risk of bias	High risk of bias	Low risk of bias
Saim et al., 1997^12^	Low risk of bias	Low risk of bias	Low risk of bias	Not applicable	High risk of bias	High risk of bias	Low risk of bias	High risk of bias
Sangeetha et al., 2014^42^	Low risk of bias	High risk of bias	Low risk of bias	Not applicable	High risk of bias	High risk of bias	High risk of bias	High risk of bias
Schaefer, 1971^43^	High risk of bias	Low risk of bias	Low risk of bias	Not applicable	High risk of bias	High risk of bias	Low risk of bias	High risk of bias
Shaw et al., 1981^44^	High risk of bias	Low risk of bias	Low risk of bias	Not applicable	High risk of bias	High risk of bias	High risk of bias	High risk of bias
Strutt et al., 2021^37^	Low risk of bias	Low risk of bias	Low risk of bias	Not applicable	High risk of bias	Low risk of bias	High risk of bias	Low risk of bias

**Table 4:** Assessment of methodological quality of case-control studies.

Author/Year	Comparability of groups other than the presence of disease in cases or the absence of disease in controls	Cases and controls appropriately matched	Criteria used for identification of cases and controls	Exposure measurement in a standard, valid and reliable way	Same exposure measurement for cases and controls	Identification of confounding factors	Strategies to deal with confounding factors	Outcomes assessment in a standard, valid and reliable way for cases and controls	Exposure period of interest long enough to be meaningful	Appropriate statistical analysis
Giuca et al., 2011^45^	Low risk of bias	Low risk of bias	Low risk of bias	Low risk of bias	Low risk of bias	High risk of bias	High risk of bias	Low risk of bias	Low risk of bias	High risk of bias
Hardani et al., 2020^47^	Unclear risk of bias	Low risk of bias	Low risk of bias	Low risk of bias	Low risk of bias	High risk of bias	Low risk of bias	Low risk of bias	Low risk of bias	Low risk of bias
Kim et al., 2010^46^	High risk of bias	Low risk of bias	Unclear risk of bias	Low risk of bias	Low risk of bias	Low risk of bias	Low risk of bias	High risk of bias	Low risk of bias	Low risk of bias
Lee et al., 2018^48^	Low risk of bias	Low risk of bias	Unclear risk of bias	Low risk of bias	Low risk of bias	High risk of bias	Low risk of bias	Low risk of bias	Low risk of bias	Low risk of bias
Megged et al., 2018^49^	High risk of bias	Low risk of bias	Low risk of bias	Low risk of bias	Low risk of bias	Low risk of bias	Low risk of bias	High risk of bias	Low risk of bias	Low risk of bias
Mumtaz et al., 2009^50^	Unclear risk of bias	Low risk of bias	Unclear risk of bias	Low risk of bias	Low risk of bias	High risk of bias	High risk of bias	Low risk of bias	Low risk of bias	High risk of bias
Nery et al., 2010^51^	Unclear risk of bias	Low risk of bias	Low risk of bias	Low risk of bias	Low risk of bias	High risk of bias	High risk of bias	Low risk of bias	Low risk of bias	High risk of bias
Pawathil & Rajamm, 2016^52^	Low risk of bias	Low risk of bias	Unclear risk of bias	Low risk of bias	Low risk of bias	High risk of bias	High risk of bias	Unclear risk of bias	Low risk of bias	High risk of bias
Ralli et al., 2011^53^	Low risk of bias	Low risk of bias	Low risk of bias	Low risk of bias	Low risk of bias	High risk of bias	High risk of bias	Low risk of bias	Low risk of bias	High risk of bias
Torretta et al., 2013^54^	High risk of bias	Low risk of bias	Unclear risk of bias	Low risk of bias	Low risk of bias	Low risk of bias	Low risk of bias	High risk of bias	Low risk of bias	Low risk of bias
Williamson et al., 1994^55^	High risk of bias	Unclear risk of bias	Low risk of bias	Low risk of bias	Low risk of bias	High risk of bias	High risk of bias	Low risk of bias	Low risk of bias	High risk of bias

**Table 5:** Assessment of methodological quality of cohort studies.

Author/Year	Similarity of groups similar and recruitment from the same population	Similar exposure measurement to assign people to both exposed and unexposed groups	Exposure measurement in a valid and reliable way	Identification of confounding factors	Strategies to deal with confounding factors	Groups/participants free of the outcome at the start of the study	Outcomes measurement in a valid and reliable way	Follow up time reported and sufficient to be long enough for outcomes to occur	Completion of follow up complete and description of reasons to losses	Strategies to address incomplete follow up	Appropriate statistical analysis
Alho et al., 1993^64^	Low risk of bias	Low risk of bias	Low risk of bias	High risk of bias	High risk of bias	High risk of bias	High risk of bias	Low risk of bias	High risk of bias	High risk of bias	High risk of bias
Bass & Groer, 1996^65^	Low risk of bias	Low risk of bias	Low risk of bias	High risk of bias	High risk of bias	Unclear risk of bias	High risk of bias	Low risk of bias	High risk of bias	High risk of bias	High risk of bias
Engel et al*.*, 1999^66^	Low risk of bias	Low risk of bias	Low risk of bias	High risk of bias	Low risk of bias	Unclear risk of bias	High risk of bias	Low risk of bias	High risk of bias	High risk of bias	Low risk of bias
Fosarelli et al., 1985^67^	Low risk of bias	Low risk of bias	Low risk of bias	High risk of bias	High risk of bias	Low risk of bias	High risk of bias	Low risk of bias	Low risk of bias	Low risk of bias	High risk of bias
Frank et al., 1982^56^	Low risk of bias	Low risk of bias	Low risk of bias	High risk of bias	High risk of bias	Unclear risk of bias	High risk of bias	Low risk of bias	High risk of bias	High risk of bias	High risk of bias
Holmes et al., 1983^57^	Low risk of bias	Low risk of bias	Low risk of bias	High risk of bias	High risk of bias	Unclear risk of bias	High risk of bias	Low risk of bias	High risk of bias	Low risk of bias	High risk of bias
Howie et al., 1990^23^	Low risk of bias	Low risk of bias	Low risk of bias	High risk of bias	Low risk of bias	Unclear risk of bias	High risk of bias	Low risk of bias	Low risk of bias	Low risk of bias	Low risk of bias
Korvel-Hanquist et al., 2018^58^	Unclear risk of bias	Low risk of bias	Low risk of bias	Low risk of bias	Low risk of bias	Unclear risk of bias	High risk of bias	Low risk of bias	High risk of bias	High risk of bias	Low risk of bias
Morin et al., 2012^59^	Low risk of bias	Low risk of bias	Low risk of bias	Low risk of bias	Low risk of bias	Low risk of bias	High risk of bias	Low risk of bias	Low risk of bias	Low risk of bias	Low risk of bias
Niemela et al., 1995^60^	Low risk of bias	Low risk of bias	Low risk of bias	Low risk of bias	Low risk of bias	Unclear risk of bias	High risk of bias	Low risk of bias	High risk of bias	High risk of bias	Low risk of bias
Rovers et al., 2008^61^	Low risk of bias	Low risk of bias	Low risk of bias	Low risk of bias	Low risk of bias	Unclear risk of bias	High risk of bias	Low risk of bias	Unclear risk of bias	High risk of bias	Low risk of bias
Saarinen, 1982^26^	Low risk of bias	Low risk of bias	Low risk of bias	High risk of bias	High risk of bias	Unclear risk of bias	High risk of bias	Low risk of bias	High risk of bias	High risk of bias	High risk of bias
Walsh et al., 2014^62^	Low risk of bias	Low risk of bias	Low risk of bias	High risk of bias	Low risk of bias	Unclear risk of bias	High risk of bias	Low risk of bias	High risk of bias	Low risk of bias	Low risk of bias
Warren et al., 2001^63^	Low risk of bias	Low risk of bias	Low risk of bias	Low risk of bias	Low risk of bias	Unclear risk of bias	High risk of bias	Low risk of bias	High risk of bias	High risk of bias	Low risk of bias

In the assessment of exposure, all studies were classified as having a low risk of bias, since the presence or absence of potentially harmful sucking habits was reported by a parent or caregiver. Currently, no validated instruments exist for the objective assessment of these habits, making reports from individuals living with the child—typically a parent or caregiver—the standard approach.

Among the 11 cross-sectional studies, seven clearly defined the inclusion criteria.^11-13,37,39,40,42^ However, only two demonstrated a low risk of bias concerning the identification of confounding factors,^38,40^ and only three adequately reported strategies for managing these confounders.^13,37,38^ Among the 11 case-control studies, only one did not provide sufficient information regarding appropriate matching of cases and controls, resulting in an unclear risk of bias.^55^ All other studies were classified as having a low risk of bias for this criterion. As with the cross-sectional studies, the identification of confounding factors was a major concern in the case-control studies. Only three studies adequately addressed confounding variables in relation to otitis media.^46,49,54^

Among the 14 cohort studies, most were considered at low risk of bias for the recruitment of groups from the same population, with only one study showing an unclear risk for this item.^58^ Many studies failed to clearly state whether participants were free of the outcome at baseline, resulting in an unclear risk of bias in 12 studies.^23,26,56^ A high risk of bias related to confounding factor identification was noted in nine studies,^23,26,56,57,62,64-67^ while five studies demonstrated a low risk of bias for this criterion.^58-61,63^

## Synthesis of results

### Otitis media and pacifier use

**Figure 2:**
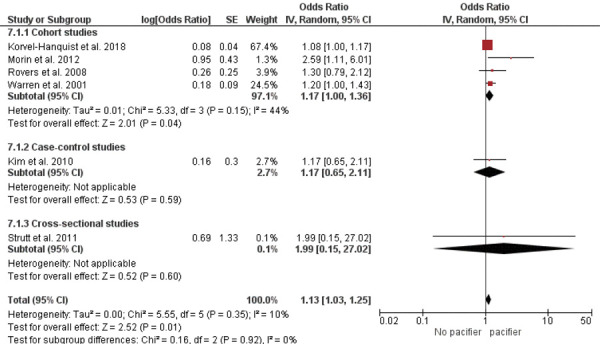
Meta-analysis of otitis media and pacifier use.

A meta-analysis of seven studies assessing otitis media (regardless of type) showed that children who used a pacifier were more likely to develop otitis media than those who did not [OR = 1.13; 95%CI: 1.03–1.25 (p = 0.01); I^2^ = 10%; chi^2^ = 5.55 (p = 0.35)] (Figure 2). The ES was 0.05 (SE = 0.01). Subgroup analysis of cohort studies also demonstrated a higher likelihood of otitis media among pacifier users [OR = 1.17; 95%CI: 1.00–1.33 (p = 0.04); I^2^ = 44%; chi^2^ = 5.33 (p = 0.15)] (Figure 2), with an ES of 0.08 (SE = 0.04). No significant difference in the occurrence of otitis media (regardless of type) was found between children who used a pacifier and those who did not in the subgroup analysis of case-control studies [OR = 1.17; 95%CI: 0.65–2.11 (p = 0.59); test of heterogeneity not applicable] (Figure 2), nor in the subgroup analysis based on the cross-sectional study [OR = 1.99; 95%CI: 0.15–27.02 (p = 0.003); test of heterogeneity not applicable] (Figure 2). The ESs were 0.08 (SE = 0.20) and 0.38 (SE = 3.78), respectively.

### Otitis media and bottle feeding

**Figure 3:**
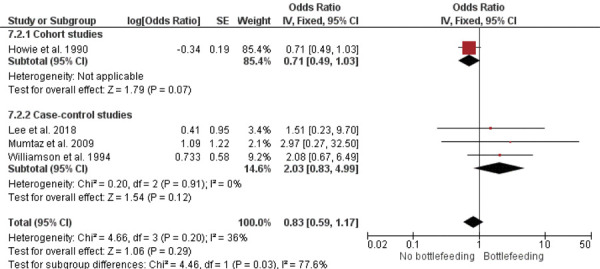
Meta-analysis of acute otitis media and bottle feeding.

A meta-analysis of four studies revealed no significant difference in the occurrence of otitis media (regardless of type) between children who were bottle-fed and those who were not [OR = 0.83; 95%CI: 0.59–1.17 (p = 0.20); I^2^ = 36%; chi^2^ = 4.66 (p = 0.20)] (Figure 3). The ES was –0.10 (SE = 0.09). Subgroup analysis of one cohort study [OR = 0.71; 95%CI: 0.49–1.03 (p = 0.07); test of heterogeneity not applicable] (Figure 3) and two case-control studies [OR = 2.03; 95%CI: 0.83–4.99 (p = 0.12); I^2^ = 0%; chi^2^=0.20 (p = 0.91)] (Figure 3), also showed no statistically significant differences. The corresponding ES values were –0.18 (SE = 0.07) and 0.39 (SE = 0.58), respectively.

### Otitis media and finger/thumb sucking

A meta-analysis could not be performed on the finger/thumb sucking habit due to insufficient data or nonstandardized data across the included studies.

### Subgroup analysis

**Figure 4:**
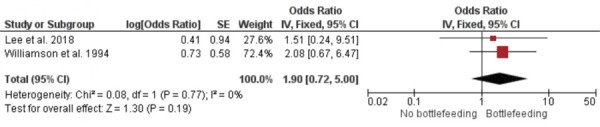
Meta-analysis of otitis media with effusion and bottle feeding (subgroup anaysis).

**Figure 5:**
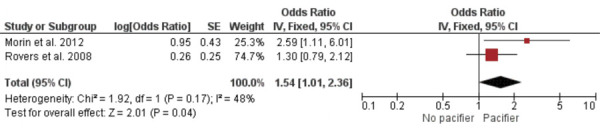
Meta-analysis of acute otitis media and pacifier use (subgroup analysis).

**Figure 6:**
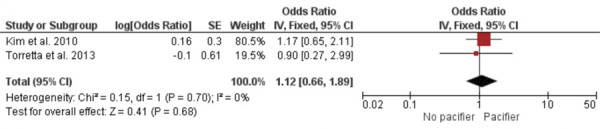
Meta-analysis of recurrent otitis media and pacifier use (subgroup analysis).

Subgroup analyses were conducted based on the specific types of otitis media associated with pacifier use and bottle feeding. A meta-analysis of two studies revealed no significant difference in otitis media with effusion between bottle-fed and non-bottle-fed children [OR = 1.90; 95%CI: 0.72–5.00 (p = 0.19); I^2^ = 0%; chi^2^ = 0.08 (p = 0.77)] (Figure 4). The ES was 0.35 (SE = 0.27). Another meta-analysis of two studies showed that children who used a pacifier were more likely to experience acute otitis media than those who did not [OR = 1.54; 95%CI: 1.01–2.36 (p = 0.04); I^2^ = 48%; chi^2^ = 1.92 (p = 0.17)] (Figure 5), with an ES of 0.23 (SE = 0.11). Finally, a meta-analysis of two studies found no significant difference in recurrent otitis media between pacifier users and non-users [OR = 1.12; 95%CI: 0.66–1.89 (p = 0.68); I^2^ = 0%; chi^2^ = 0.15 (p = 0.70)] (Figure 6). The ES was 0.06 (SE = 0.14).

### Assessment of certainty of evidence

The GRADE findings are presented in Table 6 for pacifier use (including overall otitis media, acute otitis media, and recurrent otitis media subgroups) and for bottle feeding (including overall otitis media and acute otitis media subgroups). The certainty of the evidence was rated as very low across all analyses. In general, each analysis was downgraded due to very serious concerns in the domains of risk of bias, indirectness, and imprecision. Further details are provided in the footnotes of Table 6.

**Table 6:** Summary of findings: sucking habits versus no sucking habits for otitis media (GRADE).

Certainty assessment							No. of patients		Effect		Certainty
No. of studies	Study design	Risk of bias	Inconsistency	Indirectness	Imprecision	Other considerations	Sucking habit	No sucking habit	Relative	Absolute	
									(95%CI)	(95%CI)	
Pacifier											
Otitis media											
7	Observational studies	Very serious ^a^	Serious ^b^	Very serious^c^	Serious ^d^	None	6,080 cases	9,849 controls	OR 1.11		⨁◯◯◯
							-	0.0%	(1.04, 1.19)	0 fewer per 1,000 (from 0 fewer to 0 fewer)	VERY LOW
Acute otitis media subgroup											
2	Observational studies	Very serious ^e^	Serious ^f^	Serious^g^	Very serious^h^	None		0.0%	OR 1.54	0 fewer per 1,000	⨁◯◯◯
									(1.01, 2.36)	(from 0 fewer to 0 fewer)	VERY LOW
Recurrent otitis media subgroup											
2	Observational studies	Very serious ^i^	Serious ^j^	Serious ^g^	Very serious ^h^	None	132 cases		OR 1.12	-	⨁◯◯◯
							-	0.0%	(0.66, 1.89)	0 fewer per 1,000 (from 0 fewer to 0 fewer)	VERY LOW
Bottle feeding											
Otitis media											
4	Observational studies	Very serious ^k^	Serious ^l^	Serious ^m^	Serious ^n^	None	300 cases	490 controls	OR 0.83	-	⨁◯◯◯
									(0.59, 1.17)		VERY LOW
							-	0.0%		0 fewer per 1,000 (from 0 fewer to 0 fewer)	
Acute otitis media subgroup											
2	Observational studies	Very serious ^o^	Not serious	Not serious	Very serious ^p^	None	132 cases	195 controls	OR 1.90	-	⨁◯◯◯
									(0.72, 5.00)		VERY LOW
							-	0.0%		0 fewer per 1,000 (from 0 fewer to 0 fewer)	

CI: Confidence interval; OR: Odds ratio.

a. Most studies failed to assess the outcome using valid and reliable methods and did not clearly report whether the population was free of the outcome at baseline; b. Despite low heterogeneity, there was relevant variation in effect estimates; c. Studies evaluated OM, AOM, and RAOM; results cannot be extrapolated to OME. Similarly, populations included newborns, infants, and preschoolers; results may not generalize to school-aged children; d. The number of events was adequate, but several studies had wide confidence intervals; e. Both studies failed to assess the outcome in a valid and reliable way. One also failed to address incomplete follow-up; f. Moderate heterogeneity was present among studies; g. Studies included only newborns, infants, and preschoolers, limiting generalizability to school-aged children; h. Studies had small sample sizes and wide confidence intervals; i. Both studies used inadequate outcome measures and showed a high risk of bias in the comparability of case and control groups.

j. There was relevant variation in effect estimates; k. All studies failed to identify confounding factors; some also used inadequate statistical methods; l. Moderate heterogeneity and relevant variation in estimates were observed; m. Studies evaluated OM, AOM, and OME; findings cannot be extrapolated to RAOM; n. Studies reported wide confidence intervals; o. Both studies failed to identify confounding factors; p. Studies had small sample sizes and wide confidence intervals.

## Discussion

The evidence gathered in this review indicates that children who use a pacifier are more likely to develop otitis media. Although both bottle feeding and pacifier use were initially expected to influence the occurrence of otitis media in a similar manner, no significant differences were observed between bottle-fed children and those who were not. No conclusions could be drawn regarding the association between finger/thumb sucking and otitis media.

These findings are based on meta-analyses conducted with children aged 0 to 12 years. The discrepancy between pacifier use and bottle feeding may be explained by existing literature, which suggests that the intensity, frequency, and duration of the habit are linked to the severity of its consequences.^68,69^ Pacifiers are often used for prolonged periods throughout the day, while bottle feeding typically occurs at longer intervals and shorter durations between feedings. This difference in usage patterns may contribute to the divergent results observed in the meta-analyses. Furthermore, although several studies reported an association between bottle feeding and otitis media, many were excluded from the meta-analysis due to insufficient data.

Only one study included in this review reported an association between finger/thumb sucking and the occurrence of otitis media in infants.^11^ The authors speculated that this association may be limited to children under 12 months of age; however, no additional evidence was provided to support this hypothesis. The data collected in this systematic review were insufficient to conduct a meta-analysis on finger/thumb sucking, and the theory remains unverified. Therefore, further research is warranted to clarify the relationship between finger/thumb sucking and the development of otitis media.

The findings regarding pacifier use are consistent with those of a prior meta-analytic review conducted by Uhari et al.^8^ in 1996, which also identified an increased risk of acute otitis media among pacifier users. However, the present review builds upon Uhari et al.’s work by incorporating studies published over the past two decades,^37-40,42,45-53,54,58,59,61-63^ examining a broader range of otitis media types, and including a greater number of studies overall.

According to the results of this study, children aged between 0 and 7 years who used a pacifier were 1.11 times more likely to develop otitis media. Among children aged 0 to 4 years, those who used a pacifier were 1.54 times more likely to develop acute otitis media compared to those who did not. However, it is important to emphasize that statistically significant results do not always correspond to clinical significance.^70^In this meta-analysis, ESs were close to 0.20, indicating a small effect. This suggests that the statistically significant findings may not translate into outcomes of clinical relevance.^35,71^ For a result to be considered clinically significant, three criteria must be met: a) the difference between groups must be meaningful to patients, parents (in the case of children), and professionals; b) the difference must affect an important outcome, such as symptom relief or the reduction of adverse effect; and c) the difference must be statistically significant.^68,69^In this review, 54 out of every 100 children who use a pacifier develop acute otitis media, which may substantially impact their daily lives and those of their families. Otitis media can cause discomfort, sleep disturbance, and frequent medical visits.^70^

The mechanism by which pacifier use contributes to otitis media remains uncertain. Some authors have hypothesized that potentially harmful sucking habits may interfere with Eustachian tube function.^13,74^ Pacifier use has been associated with anterior open bite and posterior crossbite,^14,15^ and studies have identified associations between these types of malocclusion and otitis media in preschool-aged children.^13,45^ However, one study that evaluated occlusal characteristics in children found no association between Eustachian tube dysfunction and anterior open bite, but rather with deep bite.^75^ Other studies have found no association between malocclusion and otitis media,^51^ and even in those that did, the relationship was not consistently linked to potentially harmful sucking habits.^45^

Alternative mechanisms should therefore be considered. Brown and Magnuson^24^ demonstrated that negative pressure is transferred to the middle ear during bottle feeding. By measuring the pressure on the bottle nipple and in the child’s ear, they found that suction creates a vacuum that generates negative pressure in the oropharynx and, subsequently, in the middle ear. This negative pressure could potentially cause the reflux of secretions into the middle ear cavity, initiating an inflammatory process.^24^ Although this review did not identify an increased risk of otitis media in bottle-fed children, it is plausible that pacifier use could similarly trigger this mechanism, potentially to an even greater extent, given the higher frequency and duration of pacifier use. Further research should explore the relationship between pacifier suction and intra-tympanic negative pressure, following a methodology similar to that used in studies on bottle feeding.

This systematic review has several strengths. We employed a comprehensive search strategy covering multiple databases and grey literature, with no restrictions on language or publication year, to ensure thoroughness. We assessed both risk of bias and certainty of the evidence, and conducted various subgroup analyses to enhance interpretability. While only observational studies were included, resulting in a lower overall level of evidence, this design is appropriate for evaluating the exposure variables of interest. Notably, most of the included studies were cohort studies, which generally provide more robust evidence compared to other observational study designs.

Many studies relied on parental reports or medical records to determine the occurrence of otitis media, which introduces a potential risk of bias due to the subjective nature of these data sources.^11,13,23,37-41,44,46,49,52,54,57-67^ Nonetheless, while some misclassification in parental reporting outcomes cannot be ruled out, it is important to recognize that parents may not seek medical care for every episode of acute ear symptoms. As a result, reliance solely on medical diagnoses could underestimate the true incidence of otitis media.^76^ Parental reporting may offer a reliable alternative and, in some cases, a more accurate measure of a child’s ear-related morbidity than clinical diagnosis. Additionally, it provides community-based data, which are highly relevant from a public health perspective^77^. For this reason, studies using parental reports were included in the present review.

However, several limitations should be acknowledged. Many of the included studies had small sample sizes, wide CIs, and a potential risk of bias. Substantial heterogeneity was also observed across studies, which may have introduced inconsistency in the results. To address this, we performed subgroup analyses. In addition, some studies lacked representation across all age groups or otitis media types, thus introducing indirectness. A further limitation was the insufficient control for confounding factors in several studies. This issue should be more rigorously addressed in future research to improve the reliability of findings. As such, caution is advised when generalizing these results. Moreover, some studies proposed evaluating key variables but failed to report corresponding data, and many provided only descriptive results. This lack of detail may suggest publication bias.

Initially, we planned to assess the prevalence of malocclusion as a secondary outcome; however, the absence of such data in most primary studies precluded this analysis. Future research should include standardized data collection on this variable. Additionally, we were unable to retrieve 23 studies identified through our search strategy. This may have limited the comprehensiveness of our review.

The comparison group for bottle-fed children is often breastfed children. However, these two feeding methods are not mutually exclusive; bottle feeding is commonly used as a complementary method. Consequently, breastfed children may also be bottle-fed. Studies that combined bottle feeding and breastfeeding in the comparison group were excluded from this review. Some studies categorized the variable as “bottle versus breast,” implying exclusivity, but this distinction was not always clearly described in the articles.

The findings of this systematic review may assist clinicians and policymakers in efforts to reduce the incidence of otitis media. While some risk factors, such as seasonal variation^78^or daycare attendance,^8^ are not easily modifiable, pacifier use should be regarded as a relevant and modifiable risk factor, with well-documented negative impacts on child health.^14,15,68^ Otitis media is a highly prevalent, multifactorial disease with substantial impact on child health. Thus, targeting avoidable exposures is essential to mitigating its incidence. Clinicians already discourage pacifier use due to its association with malocclusion and atypical swallowing, and the present review provides further support for this recommendation.

## Conclusion

In conclusion, the current scientific evidence indicates that children who use a pacifier are more likely to develop otitis media. Although this finding may have clinical relevance, it should be interpreted with caution, given that the certainty of the evidence was rated as very low according to the GRADE analysis. Future studies should adopt standardized otitis media assessment criteria and explore, in greater depth, the mechanisms by which pacifier use may contribute to the development of otitis media in children.

## Data Availability

The datasets generated during and/or analyzed during the current study are available from the corresponding author on reasonable request.
